# The characteristics of patients with multiple myeloma surviving over 10 years

**DOI:** 10.3389/fonc.2024.1490630

**Published:** 2024-11-21

**Authors:** Beihui Huang, Hongning Zhang, Junru Liu, Jingli Gu, Meilan Chen, Lifen Kuang, Xiaozhe Li, Juan Li

**Affiliations:** Department of Hematology, The First Affiliated Hospital of Sun Yat-sen University, Guangzhou, China

**Keywords:** multiple myeloma, long-term survival, prognostic factors, survival analysis, minimal residual disease

## Abstract

**Objective:**

To explore the characteristics of patients with multiple myeloma (MM) who have achieved long-term survival of over 10 years in the context where novel agents and autologous stem cell transplantation (ASCT) serve as the primary therapeutic modalities.

**Methods:**

A retrospective analysis was conducted on 168 MM patients diagnosed and treated in our institution from January 2004 to January 2014. 44 patients with a survival period exceeding 10 years were categorized into the long-term survival group, while 124 patients with a survival period of less than 10 years were categorized into the non-long-term survival group.

**Results:**

Being younger than 57 years old (OR 3.634, 95%CI 1.302-10.143), having a neutrophil count of at least 3.66 * 10^9^/L (OR 3.122, 95% CI 1.093-8.918), absence of high-risk genetic abnormalities (OR 7.146, 95%CI 1.066-47.904), and receiving frontline ASCT (OR 4.225, 95%CI 1.000-17.841) were positively associated with a survival period exceeding 10 years in patients with MM. Achieving sustained minimal residual disease (MRD) negativity for at least 24 months is associated with long-term survival regardless of the presence of high-risk cytogenetic abnormalities.

**Conclusion:**

Being younger, having a neutrophil count above 3.66 * 10^9^/L, the absence of high-risk cytogenetic abnormalities, and receiving frontline ASCT are independent protective factors for transplant-eligible MM patients to survive more than 10 years. Achieving maintained MRD negativity status for over 24 months might be associated with long-term survival.

## 1 Introduction

Multiple myeloma (MM) is a malignant plasma cell disorder primarily characterized by the clonal proliferation of abnormal plasma cells in the bone marrow, leading to the production of monoclonal proteins, or M-proteins (1). The majority of MM patients present with symptoms such as hypercalcemia, renal insufficiency, anemia, and bone destruction (1). Accounting for 1% of all neoplastic diseases, MM is the second most common hematological malignancy in high-income countries (1). As a result of the aging population and the progression of medical technology, there has been a more than twofold increase in both the incidence of MM and the number of death cases globally over the past 30 years (2).

The therapies for MM have evolved significantly over the past few decades, transitioning from conventional chemotherapy to autologous hematopoietic stem cell transplantation (ASCT) and the introduction of first- and second-generation novel agents, such as bortezomib and lenalidomide. The field is now gradually entering the era of immunotherapy. Compared to conventional chemotherapy, ASCT and treatment regimens containing agents like bortezomib and lenalidomide have significantly improved the depth of response in MM patients (3–6). These advancements in treatment approaches have led to a continuous improvement in patient prognosis (4, 7–11). However, MM remains an incurable disease, with most patients inevitably experiencing disease relapse or progression, ultimately leading to death (1). The survival for MM patients varies from a few years to several decades, and there are still few patients who can have long-term survival.

The characteristics of patients who have achieved long-term survival are of particular interest and warrant further investigation. Evangelos Terpos et al. have explored the features of MM patients with a progression-free survival (PFS) up to 7 years, identifying factors such as younger age, lower ECOG performance status, higher hemoglobin (Hb), higher creatinine clearance, International Staging System (ISS) stages I or II, normal pattern of marrow infiltration, and the absence of high-risk cytogenetics as being associated with prolonged PFS (12). However, research on the clinical characteristics of patients surviving more than 10 years is still limited.

Identifying the factors influencing long-term survival in MM patients is crucial for conducting more precise risk assessments, guiding treatment plans, and extending patient survival. Therefore, the aim of this study is to explore the clinical characteristics of patients with a survival time exceeding 10 years, as well as to describe the relationship between the minimal residual disease (MRD) evolution patterns and long-term survival by retrospectively analyzing MM patients treated at our center in recent years, particularly in the context of treatment with novel agents and ASCT.

## 2 Materials and methods

### 2.1 Patient cohorts

**Figure 1:**
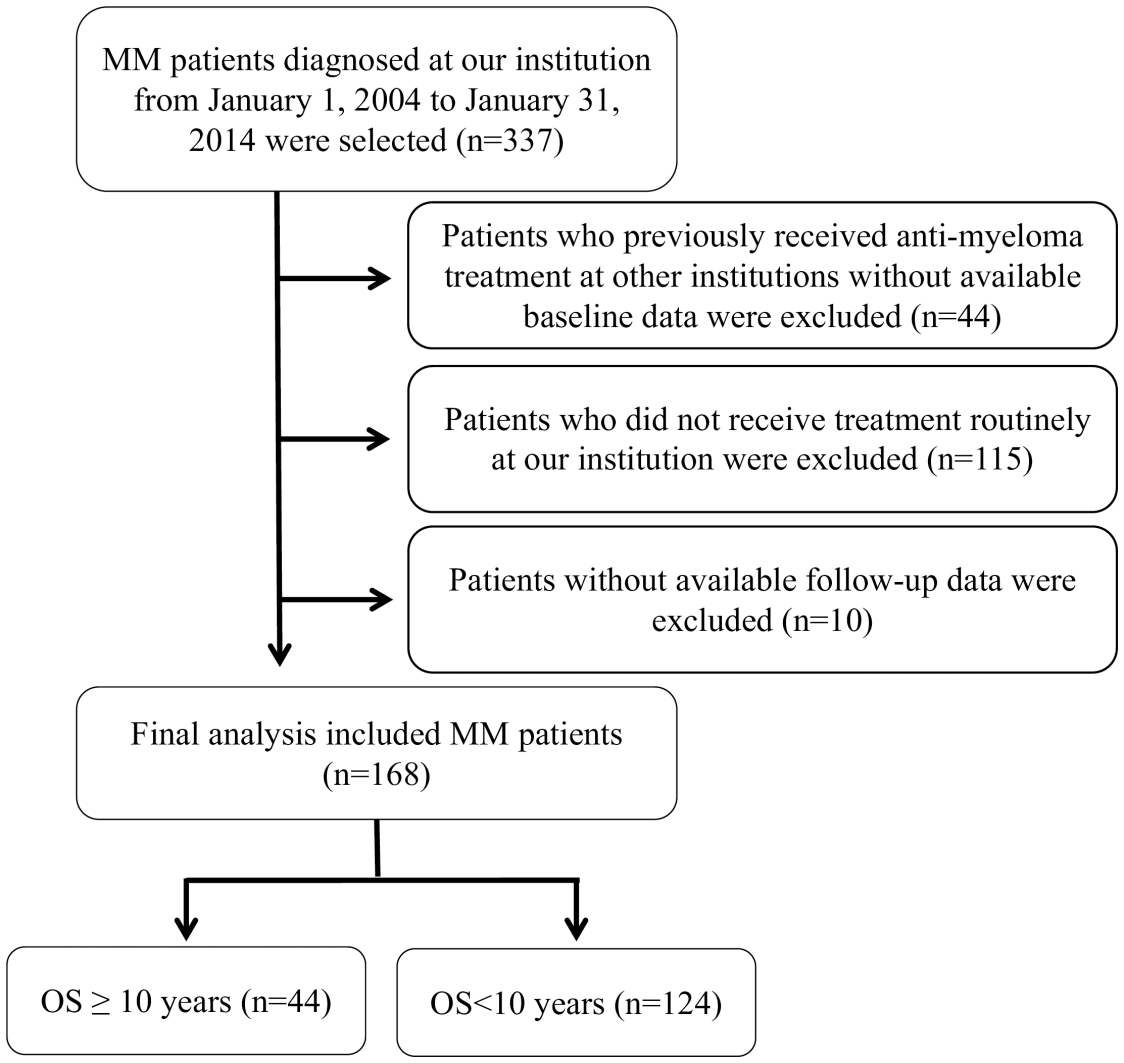
Selection and exclusion criteria for MM patients cohort analysis.

For this retrospective analysis, we included consecutively patients diagnosed with MM and treated at The First Affiliated Hospital of Sun Yat-sen University from January 1, 2004 to January 31, 2014. The exclusion criteria were as follows: (1) patients who had previously received anti-myeloma treatment at other hospitals and for whom baseline data were not available; (2) patients who did not receive routine treatment at our institution after diagnosis; (3) patients whose follow-up data was unavailable due to missing contact information or refusal of follow-up. After applying exclusion criteria, 168 MM patients with complete records on continuous treatment formed our study cohort. We defined long-term survival as an overall survival (OS) of over 10 years. Within our cohort, 44 patients (26.2%) were identified as having achieved long-term survival, while 124 patients (73.8%) did not (**Figure 1**).

### 2.2 Myeloma type, cytogenetic risk stratification and disease staging

The types of MM were determined according to the results of serum immunofixation electrophoresis. Patients were categorized into risk groups based on cytogenetic profiles identified by fluorescence *in situ* hybridization (FISH) at the time of diagnosis. High-risk was defined as the presence of any of the following chromosomal changes or their combinations: 1q gain/amplification, del17p, t (4;14), and t(14;16). Standard-risk was defined as the absence of the aforementioned chromosomal abnormalities. Patients without available cytogenetic data were categorized as other. Due to the extended period of patient enrollment, some patients lacked complete cytogenetic data, leading us to use the ISS for disease staging.

### 2.3 Treatment regimens and efficacy assessment

During the induction phase, novel agent regimens included: VD regimen (bortezomib + dexamethasone), PAD regimen (bortezomib + liposomal doxorubicin + dexamethasone), VCD regimen (bortezomib + cyclophosphamide + dexamethasone), PADT regimen (bortezomib + liposomal doxorubicin + dexamethasone + thalidomide), VTD regimen (bortezomib + thalidomide + dexamethasone), MPT regimen (melphalan + prednisone + thalidomide), CTD regimen (cyclophosphamide + thalidomide + dexamethasone), and Rd regimen (lenalidomide + dexamethasone). Conventional chemotherapy regimens included: VAD regimen (vincristine + doxorubicin + dexamethasone), VADM regimen (vincristine + doxorubicin + dexamethasone + melphalan), DVd regimen (liposomal doxorubicin + vincristine + dexamethasone), and COMP regimen (cyclophosphamide + vincristine + melphalan + prednisone).

90 patients underwent frontline ASCT with conditioning regimens including melphalan alone, bortezomib + melphalan, and the CVB regimen (cyclophosphamide + etoposide + busulfan). 118 patients received maintenance therapy including immunomodulatory drugs (IMiDs), interferons, proteasome inhibitors (PIs), or conventional chemotherapy.

Efficacy was assessed after induction and post-transplant at months 3, 6, 9, 12, 18, 24, and every 6 months thereafter according to the International Myeloma Working Group (IMWG) uniform response criteria. Responses were classified as complete remission (CR), very good partial response (VGPR), partial response (PR), minimal response (MR), stable disease (SD), and progressive disease (PD) (13).

MRD assessment, initiated from December 2010, was monitored concurrently with traditional efficacy assessment. The evaluation of bone marrow aspirate was conducted using antibody markers CD38/CD56/CD19/CD20/CD45/CD54/CD138/cκ/cλ, with a cell count threshold of 10^6^. MRD positivity was defined as the detection of abnormal phenotype plasma cells exceeding 20, with a sensitivity ranging from 2*10^-5^ to 10^-5^.

### 2.4 Follow-up

Follow-up was conducted by reviewing patients’ hospital records and through telephone contact, with the follow-up period ending in February 2024. OS was defined as the time from the start of induction therapy to death from any cause or the end of the follow-up period. PFS was defined as the time from the start of induction therapy to disease progression or death due to any reason.

### 2.5 Statistical analysis

In univariate analysis, quantitative data following a normal distribution were expressed as the mean (± standard deviation, SD), and differences between groups were compared using the independent samples t-test. For data not normally distributed, we presented the median (± interquartile range, IQR) and used the Wilcoxon rank-sum test for group comparisons. Qualitative data were expressed by frequency counts and percentages, and group differences were analyzed using the chi-square test and Fisher’s exact test when appropriate.

The Kaplan-Meier method was used to analyze survival, and differences in survival curves were assessed using the log-rank test. Variables with a P-value ≤ 0.15 in univariate analysis were included in a multivariate logistic regression model.

To facilitate clinical decision-making, continuous variables considered for inclusion in the multivariate analysis were categorized either by plotting the receiver operating characteristic (ROC) curve and determining the optimal cutoff points based on the Youden index, or by using thresholds widely accepted in clinical practice.

A P-value ≤ 0.05 was considered statistically significant. The statistical analyses and graphical representations were performed using SPSS version 25.0 and GraphPad Prism version 9.0 software, respectively.

## 3 Results

### 3.1 Baseline characteristics

A total of 168 MM patients were included in this study, with baseline characteristics presented in **Table 1**. There were 102 male patients (60.7%) and 66 female patients (39.3%), with a median age at diagnosis of 57 years (range, 28-77 years). The types of myeloma were distributed as follows: IgG in 88 cases (52.4%), IgA in 34 cases (20.2%), IgD in 9 cases (5.4%), kappa light chain type in 21 cases (12.5%), lambda light chain type in 15 cases (8.9%), and non-secretory in 1 case (0.6%).

**Table 1:** Baseline characteristics of the overall cohort at diagnosis.

Characteristic		Median (Range)/n (Ratio%)
Sex	Male	102 (60.7%)
	Female	66 (39.3%)
Age (years)		57 (28-77)
Myeloma type	IgG	88 (52.4%)
	IgA	34 (20.2%)
	IgD	9 (5.4%)
	κlight chain	21 (12.5%)
	λlight chain	15 (8.9%)
	Non-secretory	1 (0.6%)
ISS stage	I	44 (26.2%)
	II	64 (38.1%)
	III	60 (35.7%)
Ca (mmol/L)		2.40 (1.82-3.98)
CREA (umol/L)		94 (33-855)
Hb (g/L)		88 (36-175)
ALB (g/L)		36 (18-60)
LDH (U/L)		173 (58-1594)
β2MG (mg/L)		3.7 (1.1-155.7)
BMPC (%)		27.0 (1.0-89.0)
24h urinary light chain quantitation (g)		0.89 (0.00-27.60)
Cytogenetic risk	High risk	23 (13.7%)
	Standard risk	17 (10.1%)
	Other	128 (76.2%)
Induction regimens	Novel agents	128 (76.2%)
	Conventional chemotherapy	40 (23.8%)
Frontline ASCT	Yes	90 (53.6%)
	No	78 (46.4%)
Maintenance regimens	IMiDs	78 (66.1%)
	Interferon	19 (16.1%)
	IMiDs+ Interferon	14 (11.9%)
	VD	1 (0.8%)
	Conventional chemotherapy	6 (5.1%)

At diagnosis, the laboratory examination results showed a median serum total calcium concentration of 2.40 mmol/L (range, 1.82-3.98 mmol/L), a median serum creatinine (CREA) of 94 umol/L (range, 33-855 umol/L), and a median Hb of 88 g/L (range, 36-175 g/L). The median albumin (ALB) was 36 g/L (range, 18-60 g/L). The median serum β2-microglobulin (β2MG) was 3.7 mg/L (range, 1.1-155.7 mg/L), and the median lactate dehydrogenase (LDH) was 173 U/L (range, 58-1594 U/L). The median bone marrow plasma cell (BMPC) had a median level of 27.0% (range, 1.0-89.0%), and the median 24-hour urine light chain quantification was 0.89 g (range, 0.00-27.60 g). There were 44 patients (26.2%) in ISS stage I, 64 patients (38.1%) in ISS stage II, and 60 patients (35.7%) in ISS stage III at diagnosis. Among the total of 40 patients (23.8%) with complete cytogenetic data, 23 cases (13.7%) were considered as high-risk and 17 cases (10.1%) were standard-risk.

In terms of treatment, 128 patients (76.2%) received induction therapy primarily with novel agents, and 40 patients (23.8%) received induction therapy primarily with conventional chemotherapy. A total of 90 patients (53.6%) underwent frontline ASCT after induction therapy. 118 patients received maintenance therapy, consisting of 78 (66.1%) treated with single-agent immunomodulatory drugs (IMiDs), 19 (16.1%) treated with interferon monotherapy, 14 (11.9%) treated with a combination of IMiDs and interferon, 1 (0.8%) treated with bortezomib-containing maintenance therapy, and 6 (5.1%) treated with conventional chemotherapy.

In the overall study population, 19 patients (11.3%) developed a second primary malignancy (SPM), with 7 patients (15.9%) among long-term survivors and 12 patients (9.7%) among non-long-term survivors. The SPMs were predominantly hematologic malignancies, including 11 cases (57.8%) of acute lymphoblastic leukemia, 5 cases (26.3%) of myelodysplastic syndromes/acute myeloid leukemia, and 1 case (5.3%) of large granular lymphocytic leukemia. Solid tumors included 1 case (5.3%) of lung cancer and 1 case (5.3%) of nasopharyngeal tumor. Among the 19 patients who developed SPMs, 15 cases (80.0%) succumbed to it.

### 3.2 Survival and outcomes

The median follow-up time for the study population was 58.5 months (range, 0.5 to 219.4 months). The median follow-up time for the long-term survival group was 141.2 months (range, 121.0 to 219.4 months), while the median follow-up time for the non-longterm survival group was 43.1 months (range, 0.5 to 119.1 months). The median PFS for the long-term and non-long-term survival groups were 167.4 months and 24.8 months, respectively; the median OS were 192.6 months and 43.8 months, respectively.

As of the end of the follow-up period, there were 32 patients (72.7%) still alive in the long-term survival group. The most common cause of death in the long-term survival group was MM and its complications(58.3%), followed by SPMs (33.3%).

### 3.3 Characteristics of patients with survival over 10 years in the cohort

#### 3.3.1 Univariate analysis

Compared to the non-long-term survival group, the long-term survival group was significantly younger at diagnosis (median age, 50 years vs 59 years, p<0.001) and had fewer comorbidities (Charlson Comorbidity Index (CCI) <2, 86.4% vs 69.4%, p=0.027). The long-term survival group exhibited higher Hb (94g/L vs 85g/L, p=0.002) and ALB (38g/L vs 35g/L, p=0.002) than the non-long-term survival group, as well as better renal function (estimated glomerular filtration rate (eGFR), 69.58ml/min/1.73m^2^ vs 58.91ml/min/1.73m^2^, p=0.045). The proportion of patients with light chain type M-protein was higher in the long-term survival group (36.4% vs 16.1%, p=0.005). Patients in the long-term survival group more frequently had ISS stage I disease (40.9% vs 21.0%, p=0.010), serum β2MG below 5.5mg/L (79.5% vs 60.5%, p=0.022), and LDH below 260U/L (95.5% vs 82.3%, p=0.032). The proportion of patients with BMPC less than 20% was higher in the long-term survival group (52.3% vs 34.7%, p=0.040). Furthermore, the long-term survival group included a higher proportion of patients with standard-risk cytogenetic profiles (22.7% vs 5.7%, p=0.008).

In terms of treatment regimens and response, more patients in the long-term survival group received induction therapy containing novel agents (93.2% vs 70.2%, p=0.002) and frontline ASCT (84.1% vs 42.7%, p<0.001). There was also a significant difference in maintenance treatment plans between the two groups, mainly reflected in a higher proportion of patients in the long-term survival group treated with maintenance regimens containing interferon (46.5% vs 17.3%, p=0.001). Patients in the long-term survival group were more likely to achieve CR after induction (31.8% vs 13.7%, p=0.008) and post-transplant (70.3% vs 46.9%, p=0.047) compared to the non-long-term survival group. Among patients with available MRD assessment data, the proportion of patients achieving MRD negativity in the long-term survival group was higher (89.5% vs 41.8%, p<0.001), as well as the proportion of patients maintaining MRD negativity for over 24 months (97.0% vs 65.5%, p=0.001) (**Table 2**).

**Table 2:** Univariable analysis of factors affecting long-term survival.

Characteristic		Non-long-term survival group (n=124)	Long-term survival group (n=44)	P
Sex	Male	71 (57.3%)	31 (70.5%)	0.124
	Female	53 (42.7%)	13 (29.5%)	
Age (years)		59 (12)	50 (14)	<0.001
CCI	≥2	38 (30.6%)	6 (13.6%)	0.027
	<2	86 (69.4%)	38 (86.4%)	
Neutrophil (*10^9^/L)		2.84 (1.50)	3.41 (2.63)	0.086
Lymphocyte (*10^9^/L)		1.69 (1.02)	1.97 (0.82)	0.130
Hb (g/L)		85 (31)	94 (46)	0.002
PLT (*10^9^/L)		191 (111)	209 (140)	0.078
ALB (g/L)		35 (7)	38 (7)	0.002
Myeloma type 1	IgG	68 (54.8%)	20 (45.5%)	0.073
	IgA	28 (22.6%)	6 (13.6%)	
	IgD	7 (5.6%)	2 (4.5%)	
	κlight chain	10 (8.1%)	11 (25.0%)	
	λlight chain	10 (8.1%)	5 (11.4%)	
	Non-secretory	1 (0.8%)	0 (0%)	
Myeloma type 2	Light chain	20 (16.1%)	16 (36.4%)	0.005
	Non-light chain	104 (83.9%)	28 (63.6%)	
ISS stage	I	26 (21.0%)	18 (40.9%)	0.010
	II+III	98 (79.0%)	26 (59.1%)	
Ca (mmol/L)		2.40 (0.42)	2.38 (0.31)	0.423
CREA (umol/L)		95 (118)	94 (64)	0.722
eGFR (mL/min/1.73m^2^)		58.91 (53.58)	69.58 (34.44)	0.045
BMPC (%)	≥20	81 (65.3%)	21 (47.7%)	0.040
	<20	43 (34.7%)	23 (52.3%)	
β2MG (mg/L)	≤5.5	75 (60.5%)	35 (79.5%)	0.022
	>5.5	49 (39.5%)	9 (20.5%)	
LDH (U/L)	<260	102 (82.3%)	42 (95.5%)	0.032
	≥260	22 (17.7%)	2 (4.5%)	
Cytogenetic risk	High risk	19 (15.3%)	4 (9.1%)	0.008
	Standard risk	7 (5.7%)	10 (22.7%)	
	Other	98 (79.0%)	30 (68.2%)	
Induction regimens	Novel agents	87 (70.2%)	41 (93.2%)	0.002
	Conventional chemotherapy	37 (29.8%)	3 (6.8%)	
Frontline ASCT	Yes	53 (42.7%)	37 (84.1%)	<0.001
	No	71 (57.3%)	7 (15.9%)	
Maintenance	Yes	75 (74.3%)	43 (97.7%)	0.001
	No	26 (25.7%)	1 (2.3%)	
Maintenance regimens	Novel agents	59 (78.7%)	20 (46.5%)	0.001
	With interferon	13 (17.3%)	20 (46.5%)	
	Conventional Chemotherapy	3 (4.0%)	3 (7.0%)	
Response after introduction	CR	17 (13.7%)	14 (31.8%)	0.008
	Non-CR	107 (86.3%)	30 (68.2%)	
Response after ASCT	CR	23 (46.9%)	26 (70.3%)	0.047
	Non-CR	26 (53.1%)	11 (29.7%)	
Achievement of MRD negativity	Yes	33 (41.8%)	34 (89.5%)	<0.001
	No	46 (58.2%)	4 (10.5%)	
Duration of MRD negativity	≥24 months	19 (65.5%)	32 (97.0%)	0.001
	<24 months	10 (34.5%)	1 (3.0%)	

#### 3.3.2 Multivariate analysis

**Figure 2:**
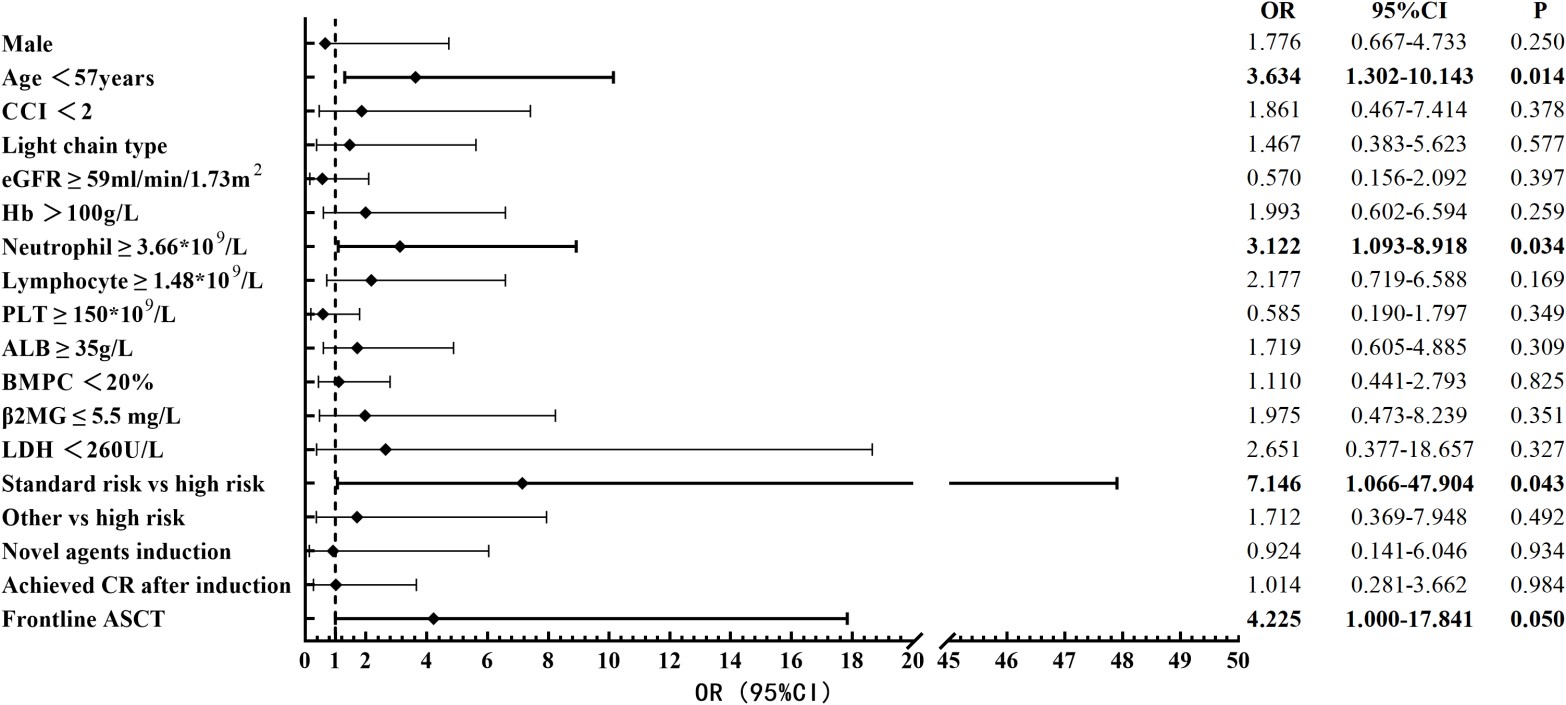
Multivariable analysis of factors affecting long-term survival. OR, odds ratio; CI, confidence interval; CCI, Charlson Comorbidity Index; eGFR, estimated glomerular filtration rate; Hb, hemoglobin; PLT, platelet count; ALB, albumin; BMPC, bone marrow plasma cells; β2MG, β2-microglobulin; LDH, lactate dehydrogenase; CR, complete response; ASCT, autologous stem cell transplantation.

We included variables with a P-value of ≤0.15 from the univariate analysis into the multivariate analysis, where variables such as age, neutrophil count, lymphocyte count, and eGFR were included in the analysis with cut-off values determined by the ROC curve (**Supplementary Figure S1**). We identified that age less than 57 years (OR 3.634, 95% CI 1.302-10.143, p=0.014), neutrophil count not lower than 3.66*10^9^/L (OR 3.122, 95% CI 1.093-8.918, p=0.034), absence of high-risk cytogenetic abnormalities (OR 7.146, 95% CI 1.066-47.904, p=0.043), and receiving frontline ASCT (OR 4.225, 95% CI 1.000-17.841, p=0.050) were independent protective factors for survival over 10 years (**Figure 2**).

### 3.4 Prognostic significance of MRD monitoring in patients with different cytogenetic status

#### 3.4.1 The prognostic significance of sustained MRD negativity for over 12 months in different cytogenetic groups

**Figure 3:**
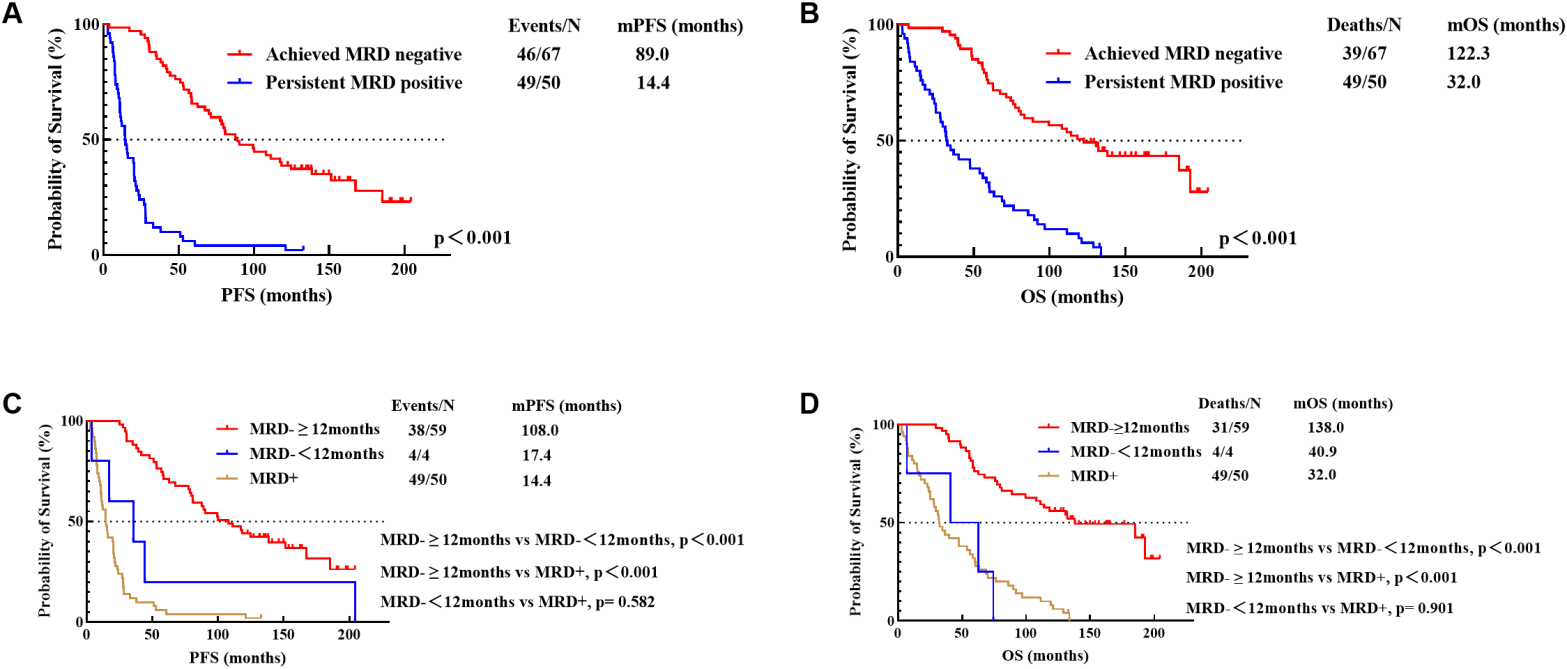
PFS **(A)** and OS **(B)** for achievement of MRD negativity and PFS **(C)** and OS **(D)** for different durations of MRD negativity (12 Months). MRD-≥12months, sustained MRD negativity for over 12 months; MRD-<12months, MRD negativity lasting less than 12 months; MRD+, persistent MRD positivity.

Firstly, among the 117 patients with available MRD monitoring data, we discovered that achieving MRD negativity during the treatment process significantly improved patients’ PFS (89.0 months vs 14.4 months, p<0.001) and OS (122.3 months vs 32.0 months, p<0.001) (**Figures 3A, B**).

Secondly, patients with MRD monitoring data were categorized into three types based on the duration of MRD negativity: those with MRD negativity sustained for over 12 months (n=59), those with MRD negativity lasting less than 12 months (n=4), and those with persistent MRD positivity (n=50). Among patients who achieved MRD negativity, the group with sustained negativity for over 12 months had significantly better PFS (108.0 months vs 17.4 months, p<0.001) and OS (138.0 months vs 40.9 months, p<0.001) compared to those with less than 12 months of negativity. While the PFS (17.4 months vs 14.4 months, p=0.582) and OS (40.9 months vs 32.0 months, p=0.901) of patients with MRD negativity lasting less than 12 months were not statistically different from those with persistent MRD positivity (**Figures 3C, D**).

Considering the analysis above, we divided the patients into two patterns based on the MRD evolution—patients who had sustained MRD negativity for over 12 months and those who did not achieve sustained MRD negativity for over 12 months (including those with MRD negativity of less than 12 months and those with persistent MRD positivity).

In high-risk patients, achieving sustained MRD negativity for over 12 months significantly improved PFS (89.0 months vs 20.4 months, p<0.001) and OS (111.2 months vs 47.6 months, p=0.050). With the exclusion of 5 patients who achieved sustained MRD negativity for over 12 months but died from SPM or myocardial infarction (where MM-related indicators did not suggest relapse or progression), the proportion of patients achieving sustained MRD negativity for over 12 months between the long-term survival group and non-long-term survival group was not statistically significant (75% vs 22%, p=0.217) (**Table 3**).

**Table 3:** Association of achieved sustained MRD negativity for over 12 months with long-term survival in high-risk and standard-risk patients.

Cytogenetic risk	MRD evolution	Non-long-term survival group	Long-term survival group	P
High risk	MRD negativity≥12months	2 (22.2%)	3 (75.0%)	0.217
	MRD negativity<12months or persistent MRD positivity	7 (77.8%)	1 (25.0%)	
Standard risk	MRD negativity≥12months	3 (100.0%)	9 (90.0%)	1.000
	MRD negativity<12months or persistent MRD positivity	0 (0%)	1 (10.0%)	

Among 16 standard-risk patients who had MRD monitoring data, 15 achieved sustained MRD negativity for over 12 months. The 15 patients had a median PFS of 78.7 months and median OS of 132.1 months. There was only 1 patient who did not achieve sustained MRD negativity for over 12 months, with an OS of 121.0 months. With the exclusion of 3 patients who achieved sustained MRD negativity for over 12 months but died from SPM (where MM-related indicators did not suggest relapse or progression), the proportion of patients achieving sustained MRD negativity for over 12 months between these two groups was also not statistically significant (90.0% vs 100.0%, p=1.000) (**Table 3**).

The above results suggest that in both standard-risk and high-risk patients, achieving sustained MRD negativity for just over 12 months may not ensure long-term survival. Therefore, we continued to explore the impact of achieving sustained MRD negativity for over 24 months on long-term survival.

#### 3.4.2 The prognostic significance of sustained MRD negativity for over 24 months in patients with different cytogenetic groups

**Figure 4:**
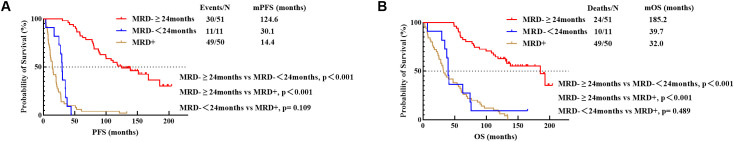
PFS **(A)** and OS **(B)** for different durations of MRD negativity (24 Months). MRD-≥24months, sustained MRD negativity for over 24 months; MRD-<24months, MRD negativity lasting less than 24 months; MRD+, persistent MRD positivity.

Patients with sustained MRD negativity for over 24 months benefited significantly with PFS (124.6 months vs 30.1 months, p<0.001) and OS (185.2 months vs 39.7 months, p<0.001) compared to those with less than 24 months of negativity. No statistically significant difference was found in either PFS (30.1 months vs 14.4 months, p=0.109) or OS (39.7 months vs 32.0 months, p=0.489) between patients with MRD negativity lasting less than 24 months and those with persistent MRD positivity (**Figures 4A, B**).

As previously described, we divided the patients into two patterns based on the MRD evolution—patients who had sustained MRD negativity for over 24 months and those who did not (including those with MRD negativity of less than 24 months and those with persistent MRD positivity).

In high-risk patients, achieving sustained MRD negativity for over 24 months significantly improved PFS (111.2 months vs 20.4 months, p<0.001) and OS (114.1 months vs 47.6 months, p=0.017). Excluding the 5 patients who achieved sustained MRD negativity for over 12 months but died from SPM or myocardial infarction (where MM-related indicators did not suggest relapse or progression), patients with sustained MRD negativity for over 24 months were more common in the long-term survival group than in the non-long-term survival group (75.0% vs 11.1%, p=0.052) (**Table 4**).

**Table 4:** Association of achieved sustained MRD negativity for over 24 months with long-term survival in high-risk and standard-risk patients.

Cytogenetic risk	MRD evolution	Non-long-term survival group	Long-term survival group	P
High risk	MRD negativity≥24months	1 (11.1%)	3 (75.0%)	0.052
	MRD negativity<24months or persistent MRD positivity	8 (88.9%)	1 (25.0%)	
Standard risk	MRD negativity≥24months	0 (0%)	9 (90.0%)	0.014
	MRD negativity<24months or persistent MRD positivity	3 (100%)	1 (10.0%)	

In standard-risk patients, sustained MRD negativity for over 24 months also significantly extended PFS (100.0 months vs 29.4 months, p=0.025) and OS (132.1 months vs 34.3 months, p=0.005). After excluding the 3 patients who achieved sustained MRD negativity for over 12 months but died from SPM (where MM-related indicators did not suggest relapse or progression), the proportion of patients with sustained MRD negativity for over 24 months was significantly higher in the long-term survival group than in the non-long-term survival group (90.0% vs 0%, p=0.014) (**Table 4**).

## 4 Discussion

Although MM remains an incurable disease, advancements in treatment have caused significant improvements in patients’ outcomes over the past few decades (4, 7–11). The treatment of MM is gradually entering the era of immunotherapy. Targeted therapies, represented by anti CD38 monoclonal antibodies (eg, daratumumab and isatuximab) and CAR-T therapies, have further improved the prognosis of MM patients, especially for those with relapsed/refractory MM (14–16). Additionally, the introduction of selective nuclear export protein inhibitors such as selinexor, second-generation proteasome inhibitors like carfilzomib, and third-generation immunomodulatory agents like pomalidomide has provided more varied treatment options (17–19). However, of the drugs mentioned above, only daratumumab has been approved in our country for newly diagnosed multiple myeloma (NDMM) patients who are ineligible for transplantation. Therefore, treatment regimens based on bortezomib, thalidomide, or lenalidomide continue to dominate the first-line recommendations in many guidelines and remain the mainstream choice for the majority of NDMM patients in our country (20).

### 4.1 Key findings and limitations

In conducting an analysis of the MM patient population mainly treated with novel agents and ASCT, we found that being younger, having a neutrophil count above 3.66 * 10^9^/L, the absence of high-risk cytogenetic abnormalities, and receiving frontline ASCT were significantly associated with survival exceeding 10 years.

It should be noted that the study has some limitations. Firstly, as a single-center study, the sample size is limited and there may be selection bias, which may affect the generalizability of the results. Secondly, we excluded patients who were missing baseline data, did not receive regular treatment, or lacked follow-up data, which might introduce bias. Thirdly, due to the delayed popularization of MRD and cytogenetic testing between 2004 and 2014, some patients lacked these important data, which limited our analysis. The sensitivity of the MRD assessment could affect the accuracy of MRD positivity determination and subsequent treatment decisions. In addition, the study involved a variety of treatment regimens, including novel agents and conventional chemotherapy, which may lead to variability in treatment responses and outcomes. The evaluation of treatment during the relapse stage was also inadequate. These limitations may restrict our in-depth understanding of the study results.

### 4.2 Clinical characteristics

In terms of clinical characteristics of MM patients, the age is undoubtedly an indispensable factor affecting survival. The median age of onset for MM patients is about 70 years (1). The IMWG has reported that for newly diagnosed eligible transplant MM patients, age over 65 years appeared to be negatively associatied with 10-year survival (21). In our study, the median age at diagnosis for long-term survivors was 50 years. Youth may indicate a better physical condition and a greater ability to withstand the disease and its treatment. In addition, ALB is an important factor affecting long-term survival. In our cohort, the median ALB level for patients surviving over 10 years was 38g/L. ALB is involved not only in the regulation of inflammation and immune responses in the body but also in the binding and delivery of drugs (22, 23). Several scoring systems that include ALB level have been widely applied in the prognostic assessment of solid tumors (24–27). In MM, ALB level is also thought to reflect the damage to the liver caused by interleukin-6 produced in the tumor microenvironment. IMWG has found that ALB level at diagnosis is one of the independent prognostic factors for MM patients and has incorporated it into the assessment to establish the ISS (28). Our study also found that a neutrophil count of at least 3.66*10^9^/L at diagnosis was significantly correlated with survival for over 10 years. This may be because neutrophil count reflects both immune function and bone marrow function (29). A lower neutrophil count at diagnosis may be associated with an increased risk of infection during treatment and limitations in drug selection (30). Many current studies have considered the neutrophil lymphocyte ratio (NLR) as an important indicator for assessing inflammatory responses in the body. Several studies have shown that an elevated NLR (NLR≥2) is associated with poor prognosis in various solid tumors (31, 32). The mechanism may involve neutrophils promoting tumor development through angiogenesis, while lymphocytes play a role in immune surveillance and eliminate tumor cells (33). There is evidence that an elevated NLR negatively impacts the OS and PFS of MM patients (33). Therefore, the neutrophil count of MM patients at diagnosis may require our attention, as both excessively high and low neutrophil count may be associated with poor prognosis.

### 4.3 Cytogenetic characteristics

Through the analysis of the cytogenetic characteristics of MM patients, we found that carrying high-risk cytogenetic abnormalities remained an independent risk factor affecting long-term survival. In our cohort, only 17.4% of patients with high-risk cytogenetic abnormalities achieved long-term survival. These patients all received a comprehensive treatment regimen that including induction with novel agents, followed by ASCT and maintenance therapy, suggesting that this treatment modality may improve the prognosis of high-risk patients. Whether tandem ASCT can overcome high-risk cytogenetic abnormalities is still controversial. According to a Canadian study, compared with a single ASCT, tandem ASCT improved the PFS of high-risk MM patients, but not OS (10); while in the EMN02/HO95 study, tandem ASCT significantly improved the survival of MM patients with high-risk cytogenetics compared to a single ASCT (34). In addition, the FORTH study showed that maintenance therapy with carfilzomib combined with lenalidomide also improved the prognosis of high-risk MM patients (35); the GMMG-CONCEPT trial reported that the quadruplet regimen of Isa-KRd (isatuximab + carfilzomib + lenalidomide + dexamethasone) achieved rapid and deep remission in high-risk MM patients, with a 2-year PFS rate of 75.5% (36). These encourage us to actively explore treatment plans suitable for high-risk MM.

### 4.4 Treatment and efficacy assessment

As for treatment, we found that receiving frontline ASCT was an independent positive factor for long-term survival in the overall cohort. Whether in the era of conventional chemotherapy or in the current era of new drugs, ASCT can bring benefits to the survival of transplant eligible MM patients, making it always considered the preferred treatment choice for newly diagnosed transplant-eligible patients (3, 37, 38). Moreover, the upper age limit for ASCT is gradually being expanded internationally (39). These demonstrate the important role of ASCT in the treatment of MM.

Our study also noted that patients who have achieved CR after induction therapy and post-transplant therapy was correlated with long-term survival, reflecting the importance of continuous and dynamic assessment of treatment response to timely adjust treatment strategies for patients with suboptimal responses to achieve deeper remission. However, even patients who have achieved CR may exhibit significant variability in survival, indicating that traditional response criteria developed by the IMWG may not fully meet current clinical needs. MRD assessment can more precisely identify the depth of disease remission (40). Analysis of MRD data in our cohorts showed that patients with sustained MRD negativity beyond 24 months generally had a better prognosis, which is consistent with our previous studies (41). Further analysis revealed that sustained MRD negativity for more than 24 months, not just over 12 months, is the critical factor associated with a survival period exceeding 10 years in patients with both high-risk and standard-risk cytogenetic profiles. This suggests that regardless of the presence of high-risk cytogenetic abnormalities, we should strive to achieve and maintain an MRD-negative state for more than 24 months to achieve the goal of long-term survival.

With the advancement of laboratory medicine, MRD detection techniques such as NGS (next-generation sequencing) and NGF (next-generation flow) have become efficient, accurate, and diverse in functionality (42). At diagnosis, NGS can detect almost all immunoglobulin gene rearrangements, including rare mutations or clonal variants that other techniques might miss, enabling risk stratification of patients (42). During treatment, NGS and NGF are not only used to assess therapeutic efficacy and monitor early relapses, but NGS also has the potential to identify drug-resistant subclones (43). These capabilities will assist in formulating and adjusting treatment plans. Furthermore, NGS provides information on clonal evolution, which helps to reveal the mechanisms of tumor development (42). Our data represents only a preliminary exploration into using MRD monitoring to guide treatment. The pursuit of more mature monitoring and treatment systems remains a topic of significant interest.

### 4.5 Clinical implications

This study conducted an analysis of MM patients who have survived over 10 years, specifically within the context where novel agents and ASCT were the primary treatments, to explore their characteristics regarding clinical features, genetic changes, treatment options, and depth of response. We were surprised to find that, despite the fact that the drugs administered to the patients in our study were not as effective as the current medications such as carfilzomib, pomalidomide, and daratumumab due to the large time span, some patients still achieved long-term survival. With the continuous emergence of a large numbers of new drugs, it’s been clearly demonstrated that quadruplet therapy containing a monoclonal antibody is superior to doublet or triplet therapies (15, 44, 45). Moreover, the early application of immunotherapy is also gradually challenging the position of ASCT. However, the induction of triplet therapy followed by ASCT remains an economically feasible and effective treatment option. The initial treatment for patients should not blindly choose potent drugs, which may also lead to the selection of drug-resistant tumor cells and higher costs. Some patients who were young at diagnosis, had sufficient neutrophil counts, and lacked high-risk cytogenetic abnormalities were very likely to achieve survival of more than 10 years through this treatment model. In cases where patients have high-risk cytogenetic abnormalities, we may incorporate CD38 monoclonal antibodies or second-generation PIs during the induction therapy phase, which may benefit patient survival (46–48). If the patient can tolerate it and has an adequate number of stem cells collected, tandem ASCT may be a good option (34). For patients with extramedullary disease, a treatment plan that includes CD38 monoclonal antibodies, second-generation PIs, third-generation IMIDs, or cytotoxic agents should be considered (49–52). Undergoing frontline ASCT can improve the poor prognosis brought by extramedullary bone related disease (EMB), while its benefits for extramedullary extraosseous disease (EME) remain controversial (53, 54). Some patients without high-risk features at initial diagnosis experience early relapse during treatment (within 12-18 months) and have a poor prognosis, which is deemed to be functional high-risk (55). These patients are primarily identified through retrospective review of medical histories, which means that early identification and preemptive interventions are difficult. However, frontline ASCT is still recommended for transplant-eligible patients. For efficacy assessment, since achieving VGPR or better before ASCT is associated with a better prognosis (56), the treatment would be adjusted if the response is suboptimal during the general induction phase. When a patient achieves VGPR after induction therapy, extending the treatment duration with the aim of achieving CR before ASCT may be considered. If a patient does not achieve VGPR after induction therapy, adjusting the treatment regimen to include drugs with different mechanisms of action or newer-generation drugs would be necessary. Once the response is improved, ASCT can then be performed. In actual clinical work, precise risk stratification to select the appropriate initial treatment and regular efficacy evaluation to timely adjust medication remain the most important topics currently.

### 4.6 Future research

At present, with the rise of immunotherapy technologies such as monoclonal antibodies, bispecific antibodies, antibody-drug conjugates, and CAR-T cell therapy, the survival prospects of MM patients have been significantly improved (57). Further studies should be multicenter and prospective, conducted in a broader patient population. They should take into account the impact of these emerging immunotherapies to fully explore the factors that may influence long-term survival, as well as the biological reasons.

### 4.7 Conclusion

In conclusion, our study identified that 26% of NDMM patients treated predominantly with novel agents and ASCT were likely to have survival exceeding 10 years. These patients were younger, exhibited a sufficient neutrophil count, were free from high-risk cytogenetic abnormalities, and had received frontline ASCT. Additionally, maintaining MRD negativity for over 24 months was also associated with long-term survival.

## Data Availability

The original contributions presented in the study are included in the article/**Supplementary Material**. Further inquiries can be directed to the corresponding author.
