# Supplementary material for: The characteristics of patients with multiple myeloma surviving over 10 years
Source: Front Oncol. 2024 Nov 21;14:1490630. doi: 10.3389/fonc.2024.1490630 (PMC11617579; doi:10.3389/fonc.2024.1490630)
Supplement: Supplementary file 1 [file DataSheet1.docx]

Supplementary Material

# Supplementary Data

Through univariate and multivariate Cox regression analysis of PFS in all patients, we found that Hb (HR 0.988, 95%CI 0.978-0.998, p=0.023), eGFR (HR 1.008, 95%CI 1.001-1.016, p=0.024), LDH (HR 1.002, 95%CI 1.000-1.003, p=0.004), and undergoing frontline ASCT (HR 0.373, 95%CI 0.220-0.633, p<0.001) were independently associated with longer PFS. (Table S2)

# Supplementary Figures and Tables

## Supplementary Figures


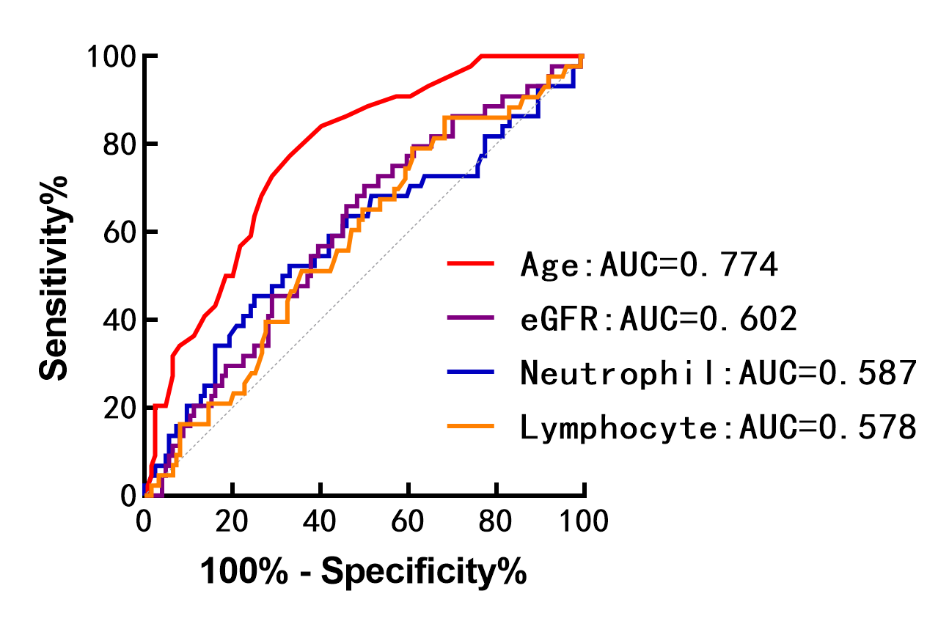


**Figure S1.** Receiver Operating Characteristic (ROC) Curve Analyses and AUC for Age, eGFR, Neutrophil Count, and Lymphocyte Count.The optimal cutoff values for age, eGFR, neutrophil count, and lymphocyte count were 57 years, 59 mL/min/1.73m^2^, 3.66 × 10^9^/L, and 1.48 × 10^9^/L, respectively. AUC, area under the curve

## Supplementary Tables

**Table S1.** Specific High-Risk Cytogenetic Abnormalities

| **High risk cytogenetic abnormalities** | | **Non-long-term survival group** | **Long-term survival group** | **P** |
| --- | --- | --- | --- | --- |
| 1q gain/amplification | Yes | 10 (50.0%) | 3 (23.1%) | 0.159 |
|  | No | 10 (50.0%) | 10 (76.9%) |  |
| t(4;14) | Yes | 4 (21.1%) | 0 (0%) | 0.119 |
|  | No | 15 (78.9%) | 14 (100.0%) |  |
| t(14;16) | Yes | 0 (0%) | 0 (0%) | - |
|  | No | 19 (100.0%) | 14 (100.0%) |  |
| del17p | Yes | 10 (15.9%) | 1 (3.7%) | 0.163 |
|  | No | 53 (84.1%) | 26 (96.3%) |  |
| High risk | Yes | 19 (73.1%) | 4 (28.6%) | 0.009 |
|  | No | 7 (26.9%) | 10 (71.4%) |  |

**Table S2.** Summary of Cox regression analysis for PFS

| **Characteristic** | **Univariate Cox regression analysis** | | **Multivariate Cox regression analysis** | |
| --- | --- | --- | --- | --- |
|  | **Hazard ratio (95%CI)** | **P** | **Hazard ratio (95%CI)** | **P** |
| Male | 0.894 (0.639-1.251) | 0.513 | - | - |
| Age (years) | 1.044 (1.025-1.063) | <0.001 | 1.017 (0.993-1.041) | 0.167 |
| CCI<2 | 0.707 (0.490-1.021) | 0.065 | 0.896 (0.574-1.4) | 0.630 |
| Light chain type | 0.567 (0.367-0.875) | 0.010 | 1.434 (0.808-2.546) | 0.219 |
| Neutrophil (*10^9^/L) | 0.959 (0.865-1.063) | 0.424 | - | - |
| Lymphocyte (*10^9^/L) | 0.910 (0.738-1.123) | 0.381 | - | - |
| Hb (g/L) | 0.983 (0.975-0.991) | <0.001 | 0.988 (0.978-0.998) | 0.023 |
| PLT (*10^9^/L) | 0.998 (0.996-1.000) | 0.058 | 0.999 (0.997-1.001) | 0.348 |
| Ca (mmol/L) | 1.492 (0.936-2.378) | 0.092 | 1.323 (0.771-2.269) | 0.310 |
| eGFR (mL/min/1.73m^2^) | 0.995 (0.990-1.000) | 0.075 | 1.008 (1.001-1.016) | 0.024 |
| ALB (g/L) | 0.962 (0.938-0.986) | 0.002 | 0.968 (0.936-1.002) | 0.063 |
| β2MG (mg/L) | 1.000 (1.000-1.000) | 0.173 | 1.000 (1.000-1.000) | 0.826 |
| LDH (U/L) | 1.002 (1.001-1.003) | <0.001 | 1.002 (1.000-1.003) | 0.004 |
| BMPC (%) | 1.011 (1.004-1.018) | 0.002 | 1.007 (0.998-1.016) | 0.155 |
| Standard risk vs high risk | 0.557 (0.272-1.141) | 0.110 | 0.653 (0.301-1.416) | 0.281 |
| Other vs high risk | 1.104 (0.685-1.780) | 0.686 | 0.834 (0.471-1.478) | 0.535 |
| Novel agents induction | 0.353 (0.242-0.516) | <0.001 | 0.634 (0.385-1.044) | 0.073 |
| Frontline ASCT | 0.391 (0.228-0.447) | <0.001 | 0.373 (0.220-0.633) | <0.001 |
| Achieve CR after induction | 0.429 (0.269-0.683) | <0.001 | 0.594 (0.343-1.030) | 0.064 |
